# Identification of the hub and prognostic genes in liver hepatocellular carcinoma *via* bioinformatics analysis

**DOI:** 10.3389/fmolb.2022.1000847

**Published:** 2022-09-29

**Authors:** Qiannan Gao, Luyun Fan, Yutong Chen, Jun Cai

**Affiliations:** ^1^ State Key Laboratory of Cardiovascular Disease, FuWai Hospital, National Center for Cardiovascular Diseases, Peking Union Medical College, Chinese Academy of Medical Sciences, Beijing, China; ^2^ Health Science Center, Peking University International Cancer Institute, Peking University, Beijing, China; ^3^ Hypertension Center, FuWai Hospital, State Key Laboratory of Cardiovascular Disease, National Center for Cardiovascular Diseases, Peking Union Medical College, Chinese Academy of Medical Sciences, Beijing, China

**Keywords:** HCC, GEO, TCGA, hub genes, prognostic model, ICGC

## Abstract

Hepatocellular carcinoma (HCC) is a common malignancy. However, the molecular mechanisms of the progression and prognosis of HCC remain unclear. In the current study, we merged three Gene Expression Omnibus (GEO) datasets and combined them with The Cancer Genome Atlas (TCGA) dataset to screen differentially expressed genes. Furthermore, protein‒protein interaction (PPI) and weighted gene coexpression network analysis (WGCNA) were used to identify key gene modules in the progression of HCC. Gene Ontology (GO) and Kyoto Encyclopedia of Genes and Genomes (KEGG) pathway enrichment analyses indicated that the terms were associated with the cell cycle and DNA replication. Then, four hub genes were identified (*AURKA, CCNB1, DLGAP5,* and *NCAPG*) and validated via the expression of proteins and transcripts using online databases. In addition, we established a prognostic model using univariate Cox proportional hazards regression and least absolute shrinkage and selection operator (LASSO) regression. Eight genes were identified as prognostic genes, and four genes (*FLVCR1, HMMR, NEB,* and *UBE2S*) were detrimental gens. The areas under the curves (AUCs) at 1, 3 and 5 years were 0.622, 0.69, and 0.684 in the test dataset, respectively. The effective of prognostic model was also validated using International Cancer Genome Consortium (ICGC) dataset. Moreover, we performed multivariate independent prognostic analysis using multivariate Cox proportional hazards regression. The results showed that the risk score was an independent risk factor. Finally, we found that all prognostic genes had a strong positive correlation with immune infiltration. In conclusion, this study identified the key hub genes in the development and progression of HCC and prognostic genes in the prognosis of HCC, which was significant for the future diagnosis and prognosis of HCC.

## Introduction

Primary liver cancer is the sixth-most frequently occurring cancer in the world and the third-most common cause of cancer mortality (Sung et al., 2021). Hepatocellular carcinoma (HCC) is the most common form of liver cancer and accounts for ∼90% of cases (Llovet et al., 2021). Although the strategy of treatment of HCC, including resection, liver transplantation, image-guided tumor ablation, image-guided transcatheter tumor therapy and systemic treatment, was effective for HCC patients, treatment indication still should be evaluated individually (Forner et al., 2018). Moreover, the methods of diagnosis of HCC remain poor except for histology for lesions and radiologic tests (Yang and Heimbach, 2020). However, some biomarkers could be utilized as diagnostic genes. For example, a set of immunostaining markers, such as glypican 3, heat shock protein 70, and glutamine synthetase, could increase diagnostic accuracy (Villanueva, 2019). Therefore, it is urgent to identify novel genes for the diagnosis of HCC and the precision medicine of HCC.

Recently, it was reported that some molecular drivers were involved in the development of HCC (Llovet et al., 2018). Studies have shown that *TERT* and *CTNNB1* mutations are associated with malignant transformation in <10% of cases (Nault et al., 2017). Other frequent mutations or genetic alterations were found in *TP53*, *RB1*, *CCNA2*, *CCNE1*, *PTEN*, *ARID1A, ARID2*, *RPS6KA3* or *NFE2L2*, all of which altered cell cycle control (Llovet et al., 2021). In addition, two major molecular subtypes of HCC were proposed (Zucman-Rossi et al., 2015). One was the proliferation gene class involved in cell proliferation and survival. It was demonstrated that *TP53* inactivation and *FGF19* and/or *CCND1* amplifications were involved (Wang et al., 2013). The other was the nonproliferation gene class, which activated the canonical *WNT* signaling pathway owing to the mutation of *CTNNB1* (Lachenmayer et al., 2012). Moreover, genome-wide gene expression studies demonstrated that some pathways, including *TGFβ*, the cell cycle, interferon, *MYC*, *PI3K/AKT,* and *MET*, were aberrantly activated in HCC (Rebouissou and Nault, 2020). Thus, it is extremely important to identify novel genes involved in the occurrence of tumors and determine the molecular mechanism of the progression of HCC.

The development of next-generation sequencing (NGS) technologies and bioinformatic tools has been widely used to search for novel targets and biomarkers for the diagnosis and precision medicine of cancer. In the current study, three Gene Expression Omnibus (GEO) datasets and The Cancer Genome Atlas (TCGA) dataset were merged and combined with bioinformatic analysis to screen differentially expressed genes (DEGs) in HCC. Then, a protein–protein interaction (PPI) network was constructed to select candidate hub genes. Moreover, DEGs of TCGA were used to screen the key gene modules using weighted gene coexpression network analysis (WGCNA). Gene Ontology (GO) and Kyoto Encyclopedia of Genes and Genomes (KEGG) pathway enrichment analyses were used to perform functional annotation and identify potential pathways in HCC, and the protein and transcript expression of hub genes was validated using the human protein atlas database and gene expression profiling interactive analysis (GEPIA) database. Additionally, univariate Cox regression analysis, LASSO regression analysis and multivariate Cox regression analysis were used to identify prognostic genes. And the effective of prognostic model also was validated using International Cancer Genome Consortium (ICGC) dataset. Overall, our work identified novel genes involved in the progression and prognosis of HCC, which was of significance for the diagnosis and treatment of HCC.

## Materials and methods

### Data collection and processing

**FIGURE 1:**
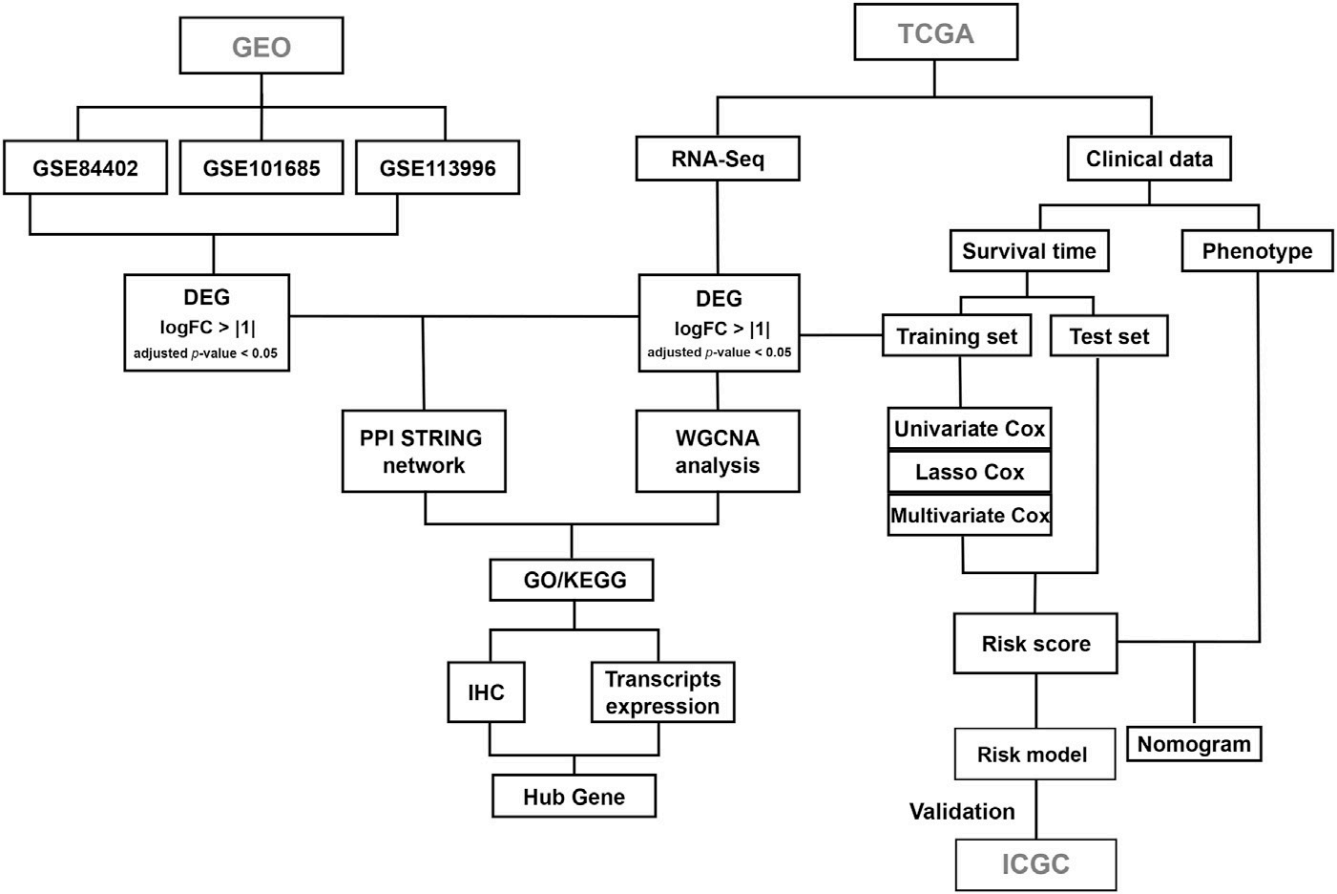
The workflow of the study design.

The workflow of the current study is shown in Figure 1. The gene expression profiles of the GSE84402, GSE101685, and GSE113996 datasets, including 42 normal samples and 58 tumor samples in total, were downloaded from the GEO database. The datasets were merged and the batch effect was eliminated using the R package “sva” (Leek et al., 2012). Differentially expressed genes (DEGs) were analyzed using the R package “limma” (Ritchie et al., 2015). *p*-value were adjusted using the false discovery rate (FDR) correction method. The cutoff for DEGs was set as |log_2_FC| > 1 and adjusted *p*-value < 0.05.

RNA sequencing (counts) and clinical data for TCGA liver hepatocellular carcinoma (LIHC) patients, including 51 normal samples and 371 tumor samples, were downloaded and analyzed using the R package “TCGAbiolinks” (Colaprico et al., 2016). The cutoff for DEGs was set as |log_2_FC| > 1 and adjusted *p*-value < 0.05. Visualization of overlapping genes in the Venn diagram was performed using the R package “VennDiagram.”

RNA sequencing (counts) and clinical data for ICGC Liver Cancer—RIKEN, JP (LIRI-JP) patients, including 232 tumor samples, were downloaded. Two samples were excluded because of infinite values in RNA sequencing data, and 230 samples were included for further analysis. LIRI-JP was used to validate the performance of the prognostic model constructed from TCGA dataset.

### Protein–protein interaction network construction and analysis of modules

DEGs were used to build a PPI network using the Search Tool for the Retrieval of Interaction Genes (STRING), and visualized using Cytoscape software. Five analysis methods in CytoHubba were used to select the key genes in PPI: edge percolated component (EPC), maximal clique centrality (MCC), maximal neighborhood component (MNC), node connect degree (degree), and node connect closeness (closeness) (Gao et al., 2020). The top 30 genes for each method were selected, and overlapping genes were identified as candidate hub genes. Molecular Complex Detection (MCODE) is a Cytoscape plugin for module analysis. Modules of interest were selected using a cutoff MCODE score >2, number of nodes >3, and confidence score >0.4.

### Weighted gene co-expression network analysis

WGCNA was used to find clusters (modules) of highly correlated genes (Langfelder and Horvath, 2008). DEGs in TCGA were subjected to WGCNA using the R package “WGCNA.” Clinical data for 421 LIHC patients were processed, with age, sex, and tumor occurrence selected as clinical traits. The soft-threshold power was used to raise the absolute value of the correlation. Hierarchical clustering and dynamic tree cut methods were used to identify modules. Eigengene networks were used to study module relationships. The module–trait relationship was assessed by Pearson’s correlation tests by attributing values of 0 and 1 to healthy individuals and tumor patients, respectively. Module membership (MM) was defined as the correlation of genes with modules of interest, where a high MMmodule score indicated that a gene was highly correlated with the module. Gene significance (GS) was defined as the correlation of genes with clinical traits. The cutoff values for hub genes were set as GS > 0.2 and MM > 0.8.

### Functional enrichment analysis

Genes in the modules of interest from WGCNA were subjected to Gene Ontology (GO) and Kyoto Encyclopedia of Genes and Genomes (KEGG) pathway enrichment analyses using The Database for Annotation, Visualization and Integrated Discovery (DAVID) database, with a comprehensive set of functional annotation tools (Dennis et al., 2003). GO terms included biological process (BP), cellular component (CC), and molecular function (MF). The cutoff was set as *p* < 0.05. Visualization of GO terms and KEGG pathways was performed using the R package “ggplot2.”

### Construction of prognostic model and survival analysis

Clinical data were downloaded using the R package “TCGAbiolinks,” and samples with missing values in terms of overall survival data were removed. Finally, a total of 364 patient samples were randomly divided into a training dataset (*n* = 273) and a test dataset (*n* = 91). In the training dataset, candidate prognostic genes were screened by univariate Cox proportional hazards regression analysis using the R package “survival.” LASSO regression analysis was used to select the prognostic gene signature (Tibshirani, 1997) using the R package “glmnet.” After performing 10-fold cross-validations 1,000 times, the minimum lambda value was confirmed. The risk score was identified as a prediction factor and calculated as follows:

$$Risk\;score=\overset{n}{\underset{i=1}{\sum }}{Coef}_{i}\times X_{i}$$

where Coef_i_ indicates the correlation coefficient of the prognostic gene signature, and X_i_ indicates the expression of the gene signature. Patients in the training and test datasets were then divided into high- and low-risk groups according to the median risk score. A heatmap of prognostic gene expression was drawn using the R package “ComplexHeatmap.” Kaplan–Meier survival curves were plotted to evaluate the predictive effect of the model using the log-rank test. The performance of the model at different endpoints (1, 3, and 5 years) was then assessed via time-dependent receiver operating characteristic (ROC) curves using the R package “timeROC.” Multivariate Cox proportional hazards regression analysis was then used to determine if the risk score and clinical information were risk factors using the R package “survminer,” with a cutoff for risk factors of *p* < 0.01. The nomogram was analyzed and depicted using the R package “nomogram.”

### Validation of hub genes and prognostic genes

The protein expression of the hub genes was validated using the Human Protein Atlas database, transcript expression of hub genes in LIHC patients was validated using the GEPIA database, and the correlation between immune infiltration and prognostic genes was validated using the Tumor Immune Estimation Resource (TIMER) database.

### Statistical analysis

Continuous variables were analyzed using Student’s *t-*test, *U-*test, or nonparametric rank-sum test. Correlations between the quantitative data were expressed by Spearman’s coefficient. Prognostic analyses were performed using univariate and multivariate Cox regression analyses. Overall survival was analyzed using Kaplan-Meier analysis, and survival differences between the high- and low-risk groups were compared by log-rank test. All statistical analyses were performed using RStudio, and *p* < 0.05 was considered significant.

## Results

### Identification of differentially expressed genes

**FIGURE 2:**
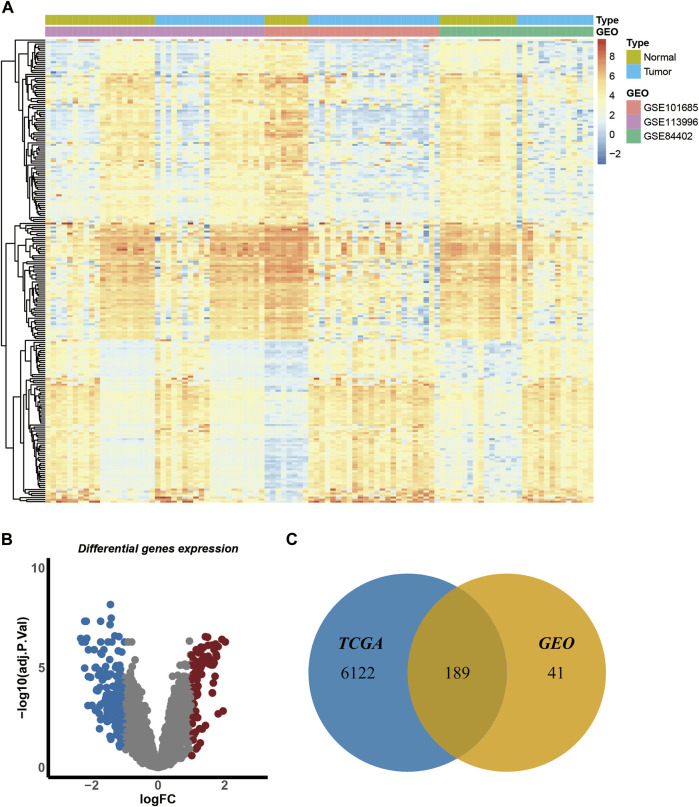
Identification of the differentially expressed genes of GEO and TCGA datasets. **(A)**. Heatmap of gene expression in merged GEO datasets. **(B)**. The volcano plot of the differentially expressed genes of merged GEO datasets. Blue dots indicate the distribution of downregulated genes, and red dots indicate the distribution of upregulated genes. **(C)**.Venn diagram of overlapping genes in GEO and TCGA datasets.

We identified DEGs in HCC by analyzing transcriptome information from three GEO datasets (GSE101685, GSE113996, and GSE84402) and TCGA (TCGA-LIHC). The GEO datasets contained 100 samples, including 42 normal and 58 tumor samples (Supplementary Table S1). The batch effect was eliminated before the analysis of DEG. A heatmap of gene expression in the merged GEO datasets is shown in Figure 2A. The criteria for the identification of DEGs were |log_2_FC| > 1 and adjusted *p*-value < 0.05. A total of 230 genes were identified, including 81 upregulated and 149 downregulated genes (Figure 2B). TCGA dataset contained 421 samples, including 50 normal and 371 tumor samples. A total of 6,311 genes were significantly expressed in TCGA dataset. Finally, a total of 189 overlapping genes were identified in the GEO and TCGA datasets (Figure 2C) and were subjected to further analyses.

### protein‒protein interaction network analysis of overlapping genes

**FIGURE 3:**
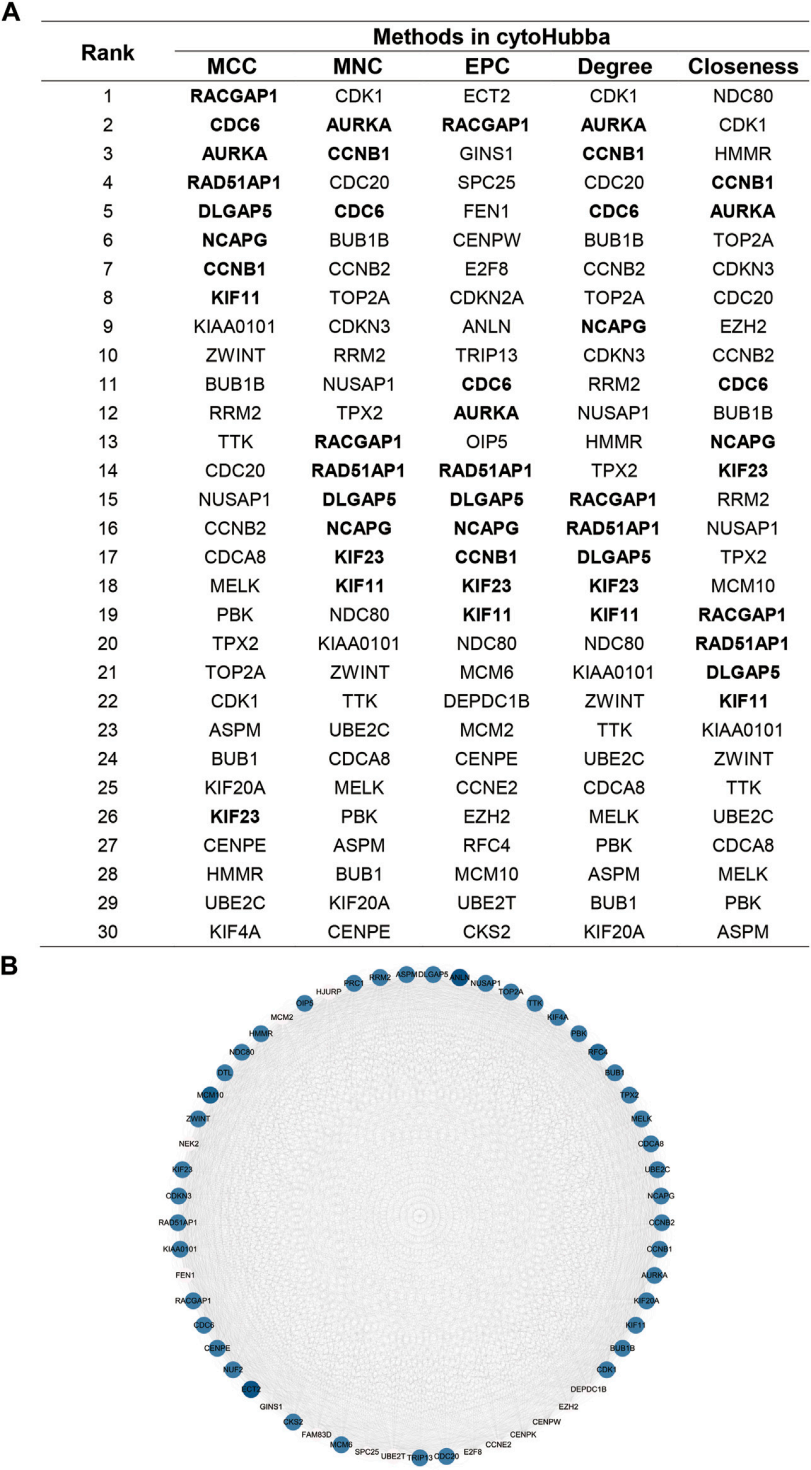
Identification of the candidate hub genes in the PPI network.**(A)**. Top 30 genes in cytoHubba.**(B)**.Gene interaction network of the most significant module in MCODE. The size of the dot is related to the degree of genes, and the gradation of the dot is related to the expression of genes.

Overlapping genes were used to perform PPI network analysis using the STRING database. The related genes were ranked and the top 30 genes were selected using five methods in cytoHubba, a plugin for rank nodes in Cytoscape. A total of nine genes overlapped and were identified as candidate hub genes (Figure 3A). Network analysis was then performed using the MCODE module in Cytoscape. The candidate hub genes were all in module 1, which was the most highly scored module (MCODE score 53.321), including 57 nodes and 2,986 edges (Figure 3B). Details of the MCODE scores are shown in Supplementary Table S2.

### Weighted gene coexpression network analysis and key module identification

**FIGURE 4:**
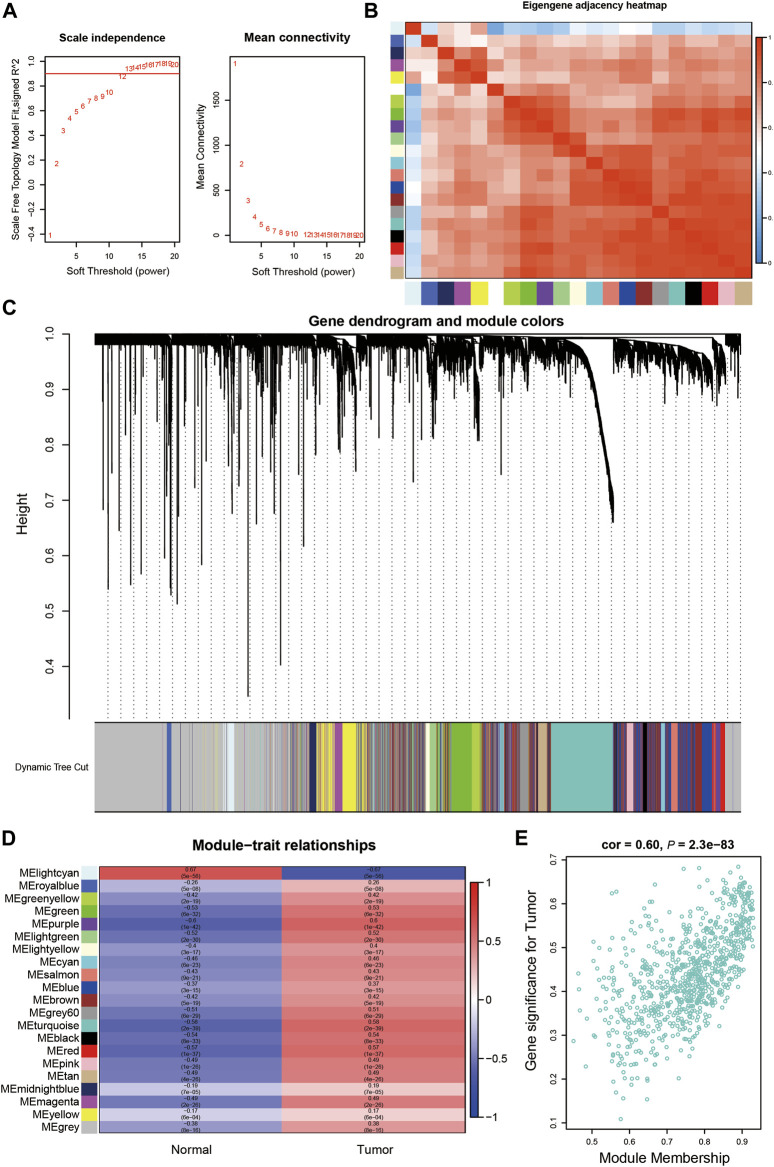
Identification of the key gene modules in WGCNA.**(A)**. Determination of the soft-thresholding power.**(B)**. The heatmap of Eigengene adjacency.**(C)**. Dendrogram of differentially expressed genes clustered based on a dissimilarity measure (1-TOM). **(D)**. The correlation of gene modules with clinical traits.**(E)** Gene correlation scatter plot of the turquoise module.

We identified the key gene modules in HCC by WGCNA using the TCGA-LIHC dataset. Samples were clustered, and TCGA.66.A9EV.01A and TCGA.DD.A3A6.01A were excluded according to their height (>160) in the hierarchical clustering tree (Supplementary Figure S1). The soft-threshold power was set as 13 based on the scale independence and mean connectivity (Figure 4A). A total of 21 modules were identified using the dynamic tree cut package (Figure 4C). The cluster of module eigengenes and the eigengene adjacency heatmap are shown in Figure 4B and Supplementary Figure S2A. We determined the correlations between the modules and the occurrence of tumors by establishing a module–trait relationship. The turquoise and purple modules (Figure 4D) were significantly correlated with tumor occurrence (coefficients 0.58 and 0.6, respectively), and the cyan module was significantly correlated with normal conditions (coefficient 0.67). In addition, high GS and high MM values were usually identified as features of hub genes. The gene distribution in the turquoise module showed that GS and MM were highly correlated, indicating that genes in this module were highly significantly associated with tumors (Figure 4E). The purple and light-cyan module are shown in Supplementary Figure S2B.

**FIGURE 5:**
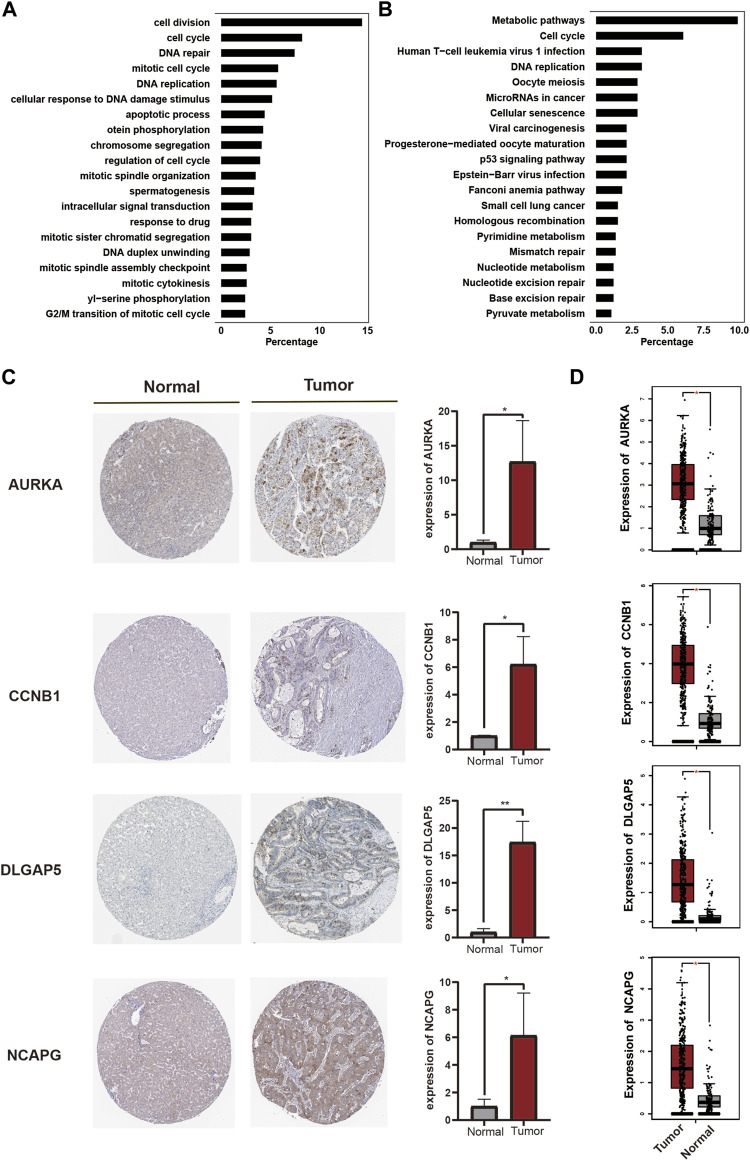
Validation of hub genes. **(A)**. GO-BP term enrichment of genes in the turquoise module. **(B)**. KEGG pathway enrichment of genes in the turquoise module. **(C)**. The protein expression of hub genes in tumor and normal samples using validation of immunohistochemistry **(D)**. Transcripts expression of hub genes in tumor and normal samples.

Genes in the turquoise module were subsequently subjected to GO and KEGG analyses. These genes were highly enriched in GO-BP terms containing cell division, cell cycle, mitotic cell cycle, and DNA replication (Figure 5A), and were enriched in GO-CC and GO-MF terms containing nucleus, cytosol, protein binding, and ATP binding (Supplementary Figure S3). KEGG pathway analysis showed that the genes were enriched in pathways including metabolic pathways, cell cycle and human T-cell leukemia virus 1 infection, and DNA replication (Figure 5B).

Moreover, the above candidate hub genes (Figure 3A) were all included in the turquoise module, suggesting that these genes played an important role in the progression of LIHC. We therefore validated the related transcript and protein expression of the candidate hub genes using the human protein atlas and Gene Expression Profiling Interactive Analysis (GEPIA) databases. Four hub genes were finally identified. The results of immunohistochemistry showed that protein expression levels of *AURKA*, *CCNB1*, *DLGAP5,* and *NCAPG* were upregulated in tumor tissue compared with normal tissue (Figure 5C). In addition, transcript levels of the four genes were also significantly upregulated in LIHC patients compared with healthy subjects (Figure 5D). The GS and MM values and the combined scores of the four hub genes are shown in Supplementary Tables S3,S4. These results thus indicated that these four genes (*AURKA*, *CCNB1*, *DLGAP5,* and *NCAPG*) were key hub genes involved in the development and progression of HCC.

### Construction of a prognostic model of Hepatocellular carcinoma

**FIGURE 6:**
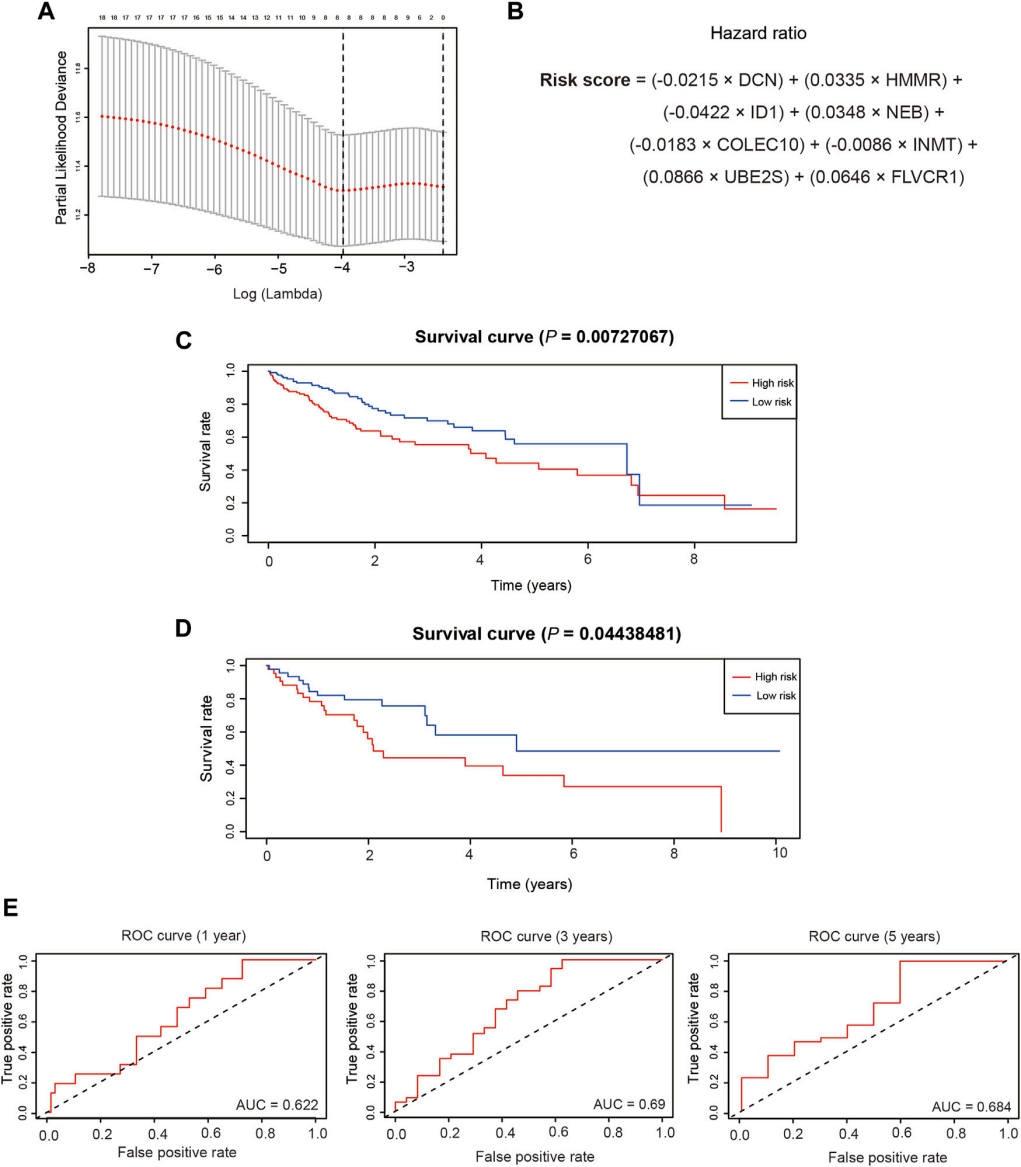
Establishment of the prognostic model. **(A)**. The selection of the minimum lambda of the lasso model via 10 folds of cross-validation. **(B)**. The calculation formula of the risk score.**(C)**.The overall survival of different risk groups in the training dataset. **(D)**. The overall survival of different risk groups in the test dataset.**(E)**. The time-dependent ROC curve of the performance of the prognostic model at 1, 3 and 5 years in the test dataset.

To establish a prognostic model of HCC, we randomly divided the subjects into a training dataset (*n* = 273) and a test dataset (*n* = 91). The training dataset was subjected to univariate Cox proportional hazards regression analysis followed by LASSO regression to screen for prognostic genes, and by multivariate Cox proportional hazards regression. A total of 19 genes were identified by univariate analysis, and LASSO regression identified eight genes with the minimum lambda value of 0.0188 (Figure 6A). The risk score was calculated as the sum of the gene coefficients multiplied by each gene expression level (Figure 6B), and the high- and low-risk patient groups were subsequently classified by the median risk score. Survival curves in the training and test datasets were examined using the Kaplan-Meier method, which showed that the low-risk group had a higher survival probability than the high-risk group in both the training and test datasets (Figures 6C,D). In addition, we predicted the overall survival in the training and test datasets at 1, 3, and 5 years. The respective areas under the time-dependent ROC curves (AUCs) were 0.622, 0.69, and 0.684 in the test dataset (Figure 6E) and 0.677, 0.645, and 0.63 in the training dataset (Supplementary Figure S4A).

**FIGURE 7:**
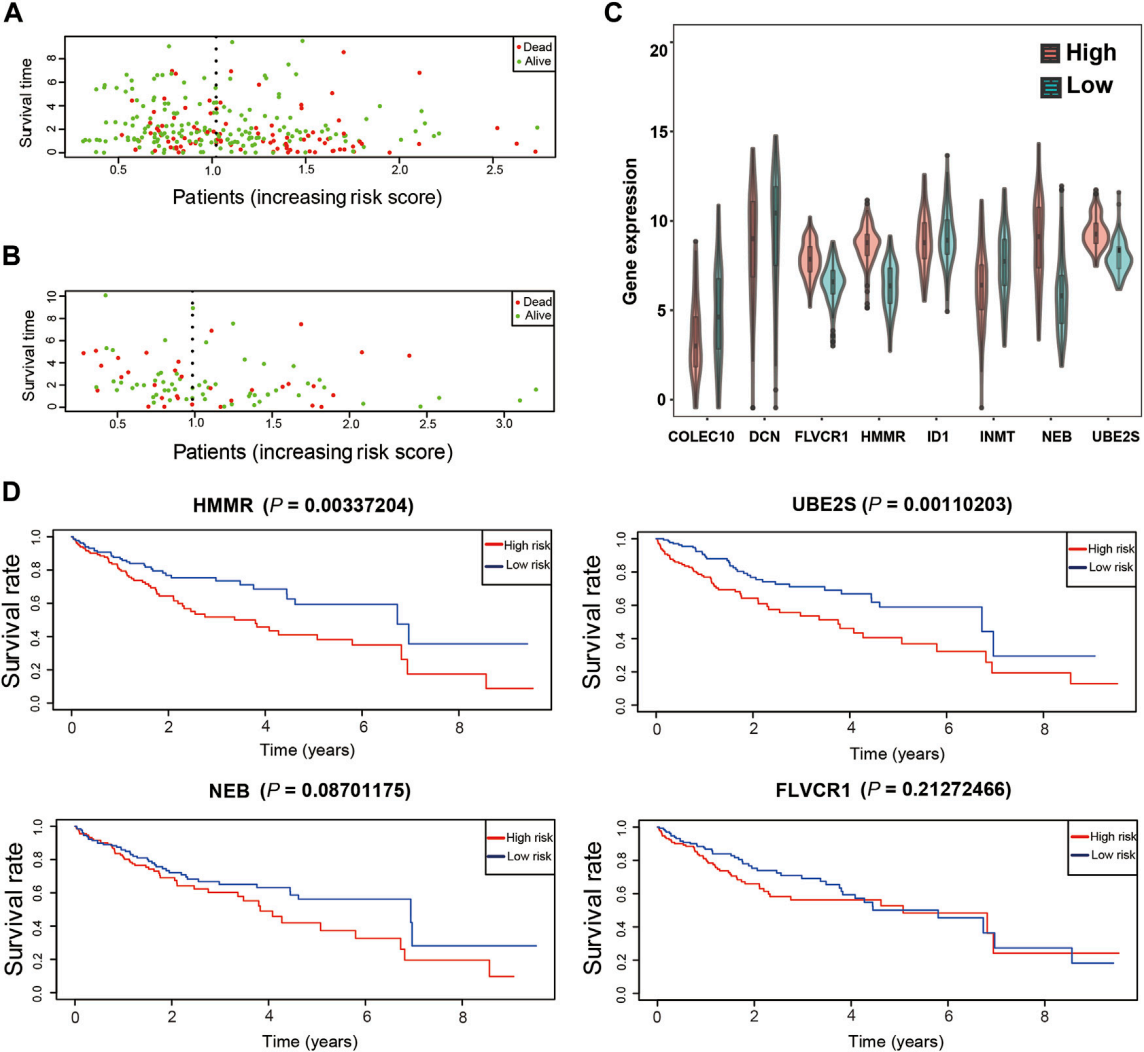
Validation of the prognostic model. **(A)**. The distribution of survival duration in the training dataset. **(B)**. The distribution of survival duration in the test dataset. **(C)**. The expression of detrimental prognostic genes in different risk groups in the training dataset. **(D)**. The overall survival of detrimental prognostic genes.

Moreover, patients with high risk scores were more likely to die in the training and test datasets (Figures 7A,B). *FLVCR1*, *HMMR*, *NEB,* and *UBE2S* expression levels were significantly upregulated in the high-risk groups compared with the low-risk groups (Figure 7C), while *COLEC10*, *DCN*, *ID1,* and *INMT* were significantly downregulated. This was in accordance with the coefficients of the LASSO model and consistent with the heatmap of gene expression in the training and test datasets (Supplementary Figure S4B). In addition, *HMMR* and *UBE2S*, but not *FLVCR1* and *NEB*, were highly associated with poor survival probability in the training dataset (Figure 7D). However, we found that only *FLVCR1* were highly associated with poor survival probability in test dataset (Supplementary Figure S4C).

**FIGURE 8:**
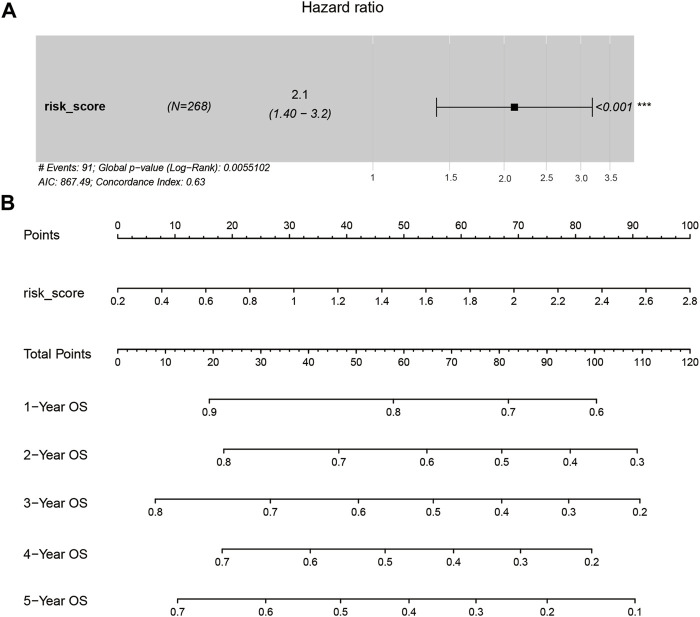
The identification of the risk score was an independent risk factor. **(A)**. The hazard ratio of risk score using multivariate Cox regression analysis. **(B)**. The nomogram of prognostic model judgment.

Multivariate Cox proportional hazards regression analysis showed that the risk score was an important factor strongly associated with the prediction of overall survival at 1, 2, 3, 4, and 5 years (Figures 8A,B). Increasing risk score was associated with a decreasing probability of overall survival in the subsequent 1–5 years. These results thus indicated that the established prognostic model could effectively predict the prognosis in patients with HCC.

### Validation of prognostic genes in International Cancer Genome Consortium dataset

**FIGURE 9:**
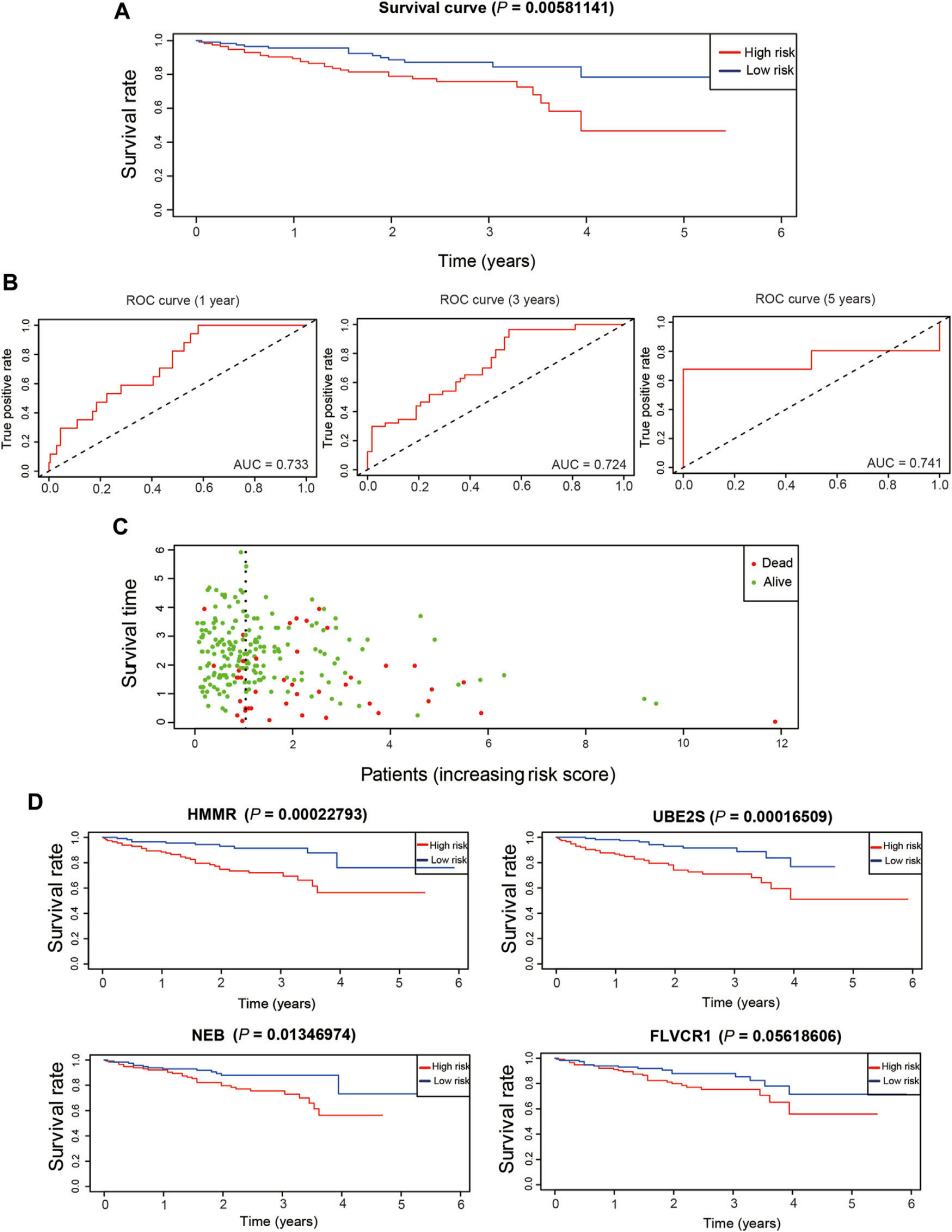
Validation of prognostic genes in ICGC dataset. **(A)**. The overall survival of different risk groups in ICGC dataset. **(B)**. The time-dependent ROC curve of the performance of the prognostic model at 1, 3 and 5 years in ICGC dataset. **(C)**. The distribution of survival duration in ICGC dataset. **(D)**. The overall survival of detrimental prognostic genes in ICGC dataset.

We validated the effectiveness of the prognostic model constructed from TCGA dataset in another dataset, LIRI-JP from the ICGC database. A total of 230 LIRI-JP RNA sequencing and clinical data were merged for further analysis. High- and low-risk patient groups were classified according to the median risk score, calculated as for the training dataset of TCGA. The low-risk group had a higher survival probability than the high-risk group (Figure 9A), and the AUCs were 0.733, 0.724, and 0.741 for predicting overall survival at 1, 3, and 5 years, respectively (Figure 9B). In addition, patients with high risk scores in the ICGC dataset were more likely to die (Figure 9C), consistent with the results for the training and test datasets. *HMMR*, *NEB*, and *UBE2S* were highly associated with poor survival probability in the ICGC dataset (Figure 9D). Taken together, our results demonstrated that the prognostic model had effective and robust performance for HCC.

### Validation of the prognostic model

**FIGURE 10:**
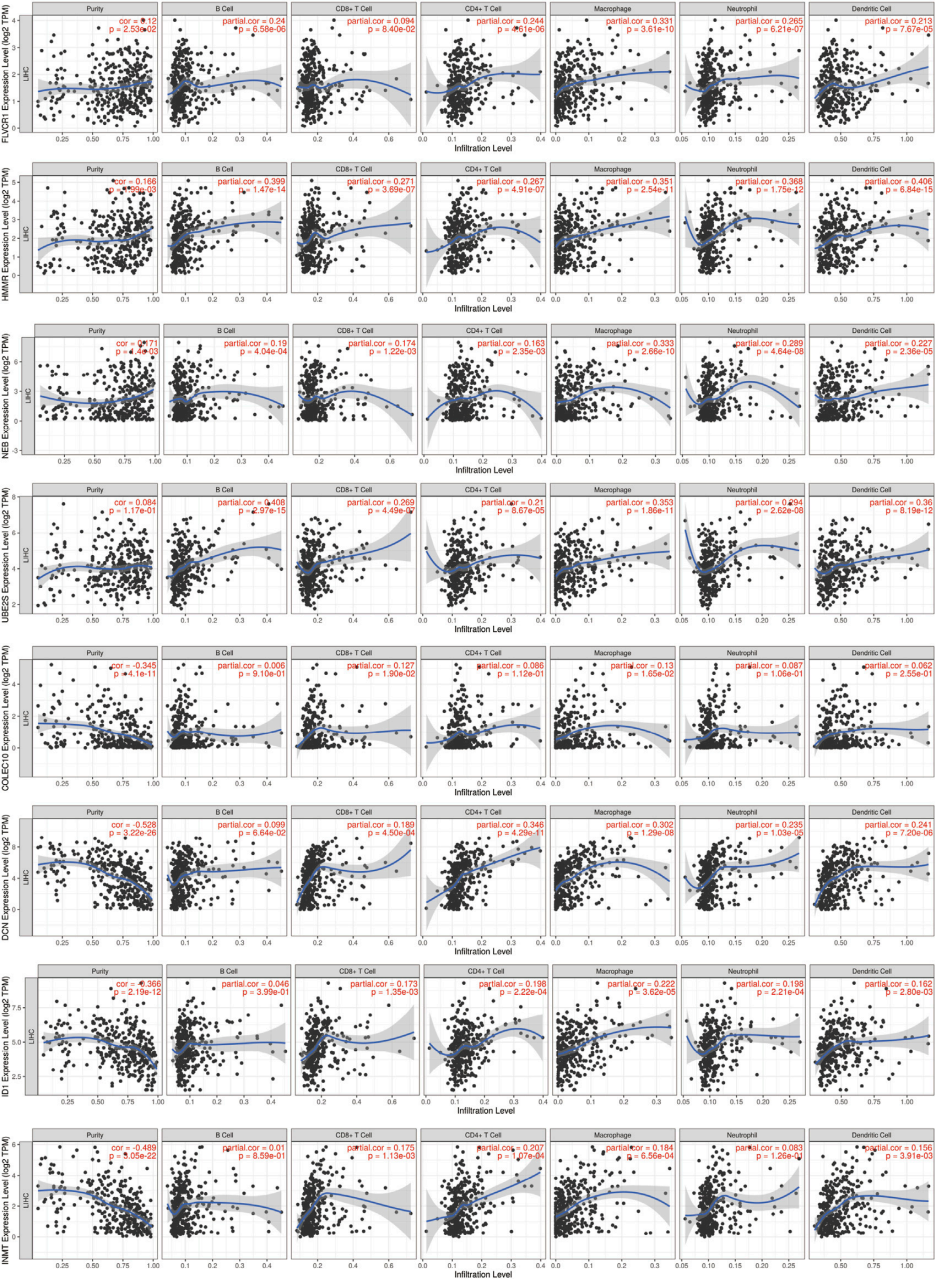
The correlation of prognostic genes with immune infiltration.

To validate the prognostic model, we analyzed the association with genes and immune infiltration. The results showed that *FLVCR1* expression was strongly positively correlated with B cells (cor = 0.24, *p* = 6.58e-06), CD4^+^ T cells (cor = 0.244, *p* = 4.61e-06), macrophages (cor = 0.331, *p* = 3.61e-10), neutrophils (cor = 0.265, *p* = 6.21e-07) and dendritic cells (cor = 0.213, *p* = 7.67e-05). *HMMR* expression was strongly positively correlated with B cells (cor = 0.399, *p* = 1.47e-14), CD8^+^ T cells (cor = 0.271, *p* = 3.69e-07), CD4^+^ T cells (cor = 0.267, *p* = 4.91e-07), macrophages (cor = 0.351, *p* = 2.54e-11), neutrophils (cor = 0.368, *p* = 1.75e-12) and dendritic cells (cor = 0.406, *p* = 6.84e-15). *NEB* expression was strongly positively correlated with B cells (cor = 0.19, *p* = 4.04e-04), CD8^+^ T cells (cor = 0.174, *p* = 1.22e-03), CD4^+^ T cells (cor = 0.163, *p* = 2.35e-03), macrophages (cor = 0.333, *p* = 2.66e-10), neutrophils (cor = 0.289, *p* = 4.64e-08) and dendritic cells (cor = 0.227, *p* = 2.63e-05). *UBE2S* expression was strongly positively correlated with B cells (cor = 0.408, *p* = 2.97e-15), CD8^+^ T cells (cor = 0.269, *p* = 4.49e-07), CD4^+^ T cells (cor = 0.21, *p* = 8.67e-05), macrophages (cor = 0.353, *p* = 1.86e-11), neutrophils (cor = 0.294, *p* = 2.62e-08) and dendritic cells (cor = 0.36, *p* = 8.19e-12). *COLEC10* expression was strongly positively correlated with CD8^+^ T cells (cor = 0.127, *p* = 1.90e-02) and macrophages (cor = 0.13, *p* = 1.65e-02). *DCN* was strongly positively correlated with CD8^+^ T cells (cor = 0.189, *p* = 4.50e-04), CD4^+^ T cells (cor = 0.346, *p* = 4.29e-11), macrophages (cor = 0.302, *p* = 1.29e-08), neutrophils (cor = 0.235, *p* = 1.03e-05) and dendritic cells (cor = 0.241, *p* = 7.20e-06). *ID1* expression was strongly positively correlated with CD8^+^ T cells (cor = 0.173, *p* = 1.35e-03), CD4^+^ T cells (cor = 0.198, *p* = 2.22e-04), macrophages (cor = 0.222, *p* = 3.62e-05), neutrophils (cor = 0.198, *p* = 2.21e-04) and dendritic cells (cor = 0.162, *p* = 2.80e-03). *INMT* expression was strongly positively correlated with CD8^+^ T cells (cor = 0.175, *p* = 1.13e-03), CD4^+^ T cells (cor = 0.207, *p* = 1.07e-04), macrophages (cor = 0.184, *p* = 6.56e-04) and dendritic cells (cor = 0.156, *p* = 3.91e-03) (Figure 10). Thus, the results above indicated that the prognostic model we established had potential and effective prediction for the prognosis of HCC.

## Discussion

In the current study, we merged three GEO datasets and TCGA datasets and combined them with bioinformatics analysis to screen and identify hub genes and prognostic genes in the development and progression of HCC. We identified four hub genes (*AURKA, CCNB1, DLGAP5,* and *NCAPG*) using WGCNA and PPI network analysis based on the clustering of the key modules and biological functional annotation. Moreover, we established a prognostic model and identified four detrimental prognostic genes (*FLVCR1, HMMR, NEB,* and *UBE2S*) using Lasso-Cox regression. Therefore, these genes could be potential biomarkers and prediction factors in the future diagnosis and treatment of HCC.

Correlation networks are increasingly being used in bioinformatics applications. WGCNA is a systems biology method for exploring the module structure in a network, measuring the relationships between genes and modules and the relationships among modules (Langfelder and Horvath, 2008). Using WGCNA, we found that the turquoise module was strongly positively correlated with the occurrence of tumors, and most genes in the turquoise module overlapped with those in the PPI networks, which indicated that these genes were highly associated with the development of HCC. Finally, we identified four hub genes (*AURKA, CCNB1, DLGAP5,* and *NCAPG*) and validated them in HCC samples.

Aurora A (*AURKA*) is a type of Aurora kinase that belongs to the family of serine/threonine kinases and plays essential roles in regulating cell division during mitosis. It was reported that the expression of *AURKA* was aberrantly high in HCC (Du et al., 2021). Increased *AURKA* expression and a positive correlation between *AURKA* and *MYC* expression were found in *TP53*-mutated human HCCs (Dauch et al., 2016), which indicated that *AURKA* was a potential druggable target. Additionally, overexpression of Aurora-A was associated with high-grade (grade II-IV) and high-stage (stage IIIB-IV) tumors, *p53* mutation, infrequent beta-catenin mutation, and poor outcome (Jeng et al., 2004).

Cyclin B1 (*CCNB1*) is a regulator involved in mitosis. *MYC* was reported to activate *WDR4* transcription, and *WDR4* promoted *CCNB1* mRNA stability and translation to enhance HCC progression (Xia et al., 2021). In addition, *CCNB1* could be a candidate biomarker and potential therapeutic target for HBV-related HCC recurrence after surgery (Weng et al., 2012). DLG-associated protein 5 (*DLGAP5*), also known as *HURP*, can enable microtubule binding activity. The molecular mechanism of *DLGAP5* in the development of HCC is limited, but known studies have reported that *DLGAP5* may be a potential biomarker in HCC (Hao et al., 2021). Non-SMC condensin I complex subunit G (*NCAPG*) was first isolated from HeLa cell nuclei and demonstrated to regulate the location of DNA on chromosomes (Sun et al., 2022). It was demonstrated that *NCAPG* was a novel mitotic gene involved in the proliferation and migration of HCC cells (Zhang et al., 2018; Gong et al., 2019). Moreover, *AURKA* and *DLGAP5* were mitosis-associated genes. *DLGAP5* is also a substrate of *AURKA* (Wu et al., 2013), which is consistent with our results that *AURKA* is a hub gene. Although investigation of the association between these four hub genes was currently poor, they were involved in the regulation of cell proliferation and cell cycle and played important role in the development and progression of cancer.

Moreover, we established the prognostic model using Lasso Cox regression and identified eight prognostic genes, four of which were detrimental genes (*NEB, UBE2S, FLVCR1,* and *HMMR*). These four genes could predict the overall survival time in the high-risk groups and low-risk groups. In addition, the prognostic model showed excellent prediction performance. Therefore, four detrimental genes could be potential diagnostic and prognostic genes.

Nebulin (*NEB*) encodes a giant protein component of the cytoskeletal matrix that coexists with the thick and thin filaments within the sarcomeres of skeletal muscle. It was reported that the mutation of *NEB* was associated with many diseases, such as nemaline myopathy (Piga et al., 2016) and fetal akinesia deformation sequence/arthrogryposis multiplex congenita (Feingold-Zadok et al., 2017). Additionally, the mutation frequency of *NEB* at the amino acid 1,133 locus of thyroid cancer patients was much higher than that of the normal population (Wang et al., 2020). Although the relationship between *NEB* and HCC has not yet been reported, further research is needed to determine the molecular mechanism of *NEB* in HCC.

Ubiquitin conjugating enzyme E2S (*UBE2S*), also known as *E2EPF*, belongs to the E2 family of proteins and elongates the K11-linked polyubiquitin chain on APC/C substrates for 26 S proteasome-mediated degradation to promote cell division (Wu et al., 2010). *UBE2S* can promote the progression of many types of cancer, such as ovarian cancer (Hu et al., 2021), non-small cell lung cancer (Qin et al., 2020), colorectal cancer (Li et al., 2018) and prostate cancer (Peng et al., 2022). In addition, *UBE2S* promoted cell chemoresistance through *PTEN-AKT* signaling in HCC (Gui et al., 2021), which indicated that *UBE2S* may be a novel oncogene in the development of cancer. Feline leukemia virus subgroup C receptor 1 (*FLVCR1*) has been reported to have a crucial role in cell proliferation and cell death (Peng et al., 2018). A recent study showed that *FLVCR1* was significantly higher in the HCC cohort from ICGC than in normal samples (Tang et al., 2020), which was consistent with our results. Hyaluronan-mediated motility receptor (*HMMR*) is an oncogene involved in neoplastic progression of human leukemias and solid tumors (Tilghman et al., 2014). The overexpression of HMMR was strongly associated with the occurrence of HCC.

However, there are some limitations in this study. First, gene-based markers as biologic signatures were not enough to use as prognostic model for predicting patient outcomes. Network or subnetworks markers need to be developed to perform more meaningful and accurate prediction. Song et al. developed a method that identified survival prognostic subnetwork markers (SPNs), which had more accurate and effective performance for prediction of distant metastasis-free survival time and uncovered the biological mechanism in in breast cancer (Song et al., 2015). Additionally, Discrepancy of tumor immune microenvironment under differed treatments need to be resolved at single-cell level. It was reported that single-cell multi-omics gene co-regulatory algorithm (SMGR) was developed to discover cis-element elements and regulatory networks in mixed-phenotype acute leukemia cells by integrating single-cell RNA-sequencing and single-cell assay for transposase-accessible chromatin using sequencing (Song et al., 2022). Taken together, more comprehensive models and integrating methods need to be used for the validation and analysis of results.

In conclusion, our results indicated that *AURKA, CCNB1, DLGAP5,* and *NCAPG* were key hub genes and that *NEB, UBE2S, FLVCR1* and *HMMR* were crucial detrimental prognostic genes, which could be potential biomarkers and druggable targets in the diagnosis and treatment of HCC.

## Data Availability

The original contributions presented in the study are included in the article/Supplementary Material, further inquiries can be directed to the corresponding author.
