# Supplementary material for: Identification of the hub and prognostic genes in liver hepatocellular carcinoma via bioinformatics analysis
Source: Front Mol Biosci. 2022 Sep 29;9:1000847. doi: 10.3389/fmolb.2022.1000847 (PMC9557295; doi:10.3389/fmolb.2022.1000847)
Supplement: Supplementary file 1 [file DataSheet1.ZIP › revised-Supplementary Materials Presentation/revised-Supplementary material.docx]

**Supplementary material**

The number of Supplementary Figures: 4

The number of Supplementary Tables: 4

**Supplementary Figure Legends**

Supplementary Figure 1. The dendrogram of cluster in TCGA samples.

Supplementary Figure 2. Identification of key modules using WGCNA.

(A). The dendrogram of the clustered modules.

(B). Gene correlation scatter plot of the lightcyan module and purple module.

Left panel: lightcyan module. Right panel: purple module.

Supplementary Figure 3. GO-CC and GO-MF terms enrichment of genes in key module.

(A). GO-CC terms enrichment of genes in the turquoise module.

(B). GO-MF terms enrichment of genes in the turquoise module.

Supplementary Figure 4. Validation of the prognostic model.

(A). The ROC curve of the performance of the prognostic model at 1, 3 and 5 years in the training dataset.

(B). Heatmap of gene expression of prognostic genes in the training and test dataset.

Left panel: training dataset. Right panel: test dataset.

(C). The overall survival of detrimental prognostic genes in test dataset.

**Supplementary Table**

Supplementary Table 1. Detail information in GEO datasets.

Supplementary Table 2. Detail information of MCODE score.

Supplementary Table 3. Value of GS and MM in four hub genes.

Supplementary Table 4. All scores by different methods of hub genes

Supplementary Table 1. Detail information in GEO datasets

| GEO accession | platform | Tumor | Normal |
| --- | --- | --- | --- |
| GSE84402 | GPL570 | 14 | 14 |
| GSE101685 | GPL570 | 24 | 8 |
| GSE101685 | GPL16043 | 20 | 20 |
| In total |  | 58 | 42 |

Supplementary Table 2. Detail information of MCODE score

| Module | nodes | edges | score |
| --- | --- | --- | --- |
| Modul1 | 57 | 2986 | 53.321 |
| Modul2 | 10 | 76 | 8.444 |
| Modul3 | 12 | 58 | 5.273 |
| Modul4 | 5 | 20 | 5.000 |
| Modul5 | 4 | 12 | 4 |

Supplementary Table 3. Value of GS and MM in four hub genes

| Gene | GS | MM |
| --- | --- | --- |
| *AURKA* | 0.624440434 | 0.819895385 |
| *CCNB1* | 0.61699264 | 0.916708944 |
| *DLGAP5* | 0.609786183 | 0.906359812 |
| *NCAPG* | 0.39858859 | 0.838999704 |

| Gene | MCC | MNC | EPC | Degree | Closeness |
| --- | --- | --- | --- | --- | --- |
| *AURKA* | 2.55E+53 | 60 | 106.542 | 120 | 93.86785714 |
| *CCNB1* | 2.55E+53 | 60 | 106.542 | 120 | 93.86785714 |
| *DLGAP5* | 2.55E+53 | 56 | 106.542 | 112 | 89.16785714 |
| *NCAPG* | 2.55E+53 | 56 | 106.542 | 114 | 89.91785714 |

Supplementary Table 4. All scores by different methods of hub genes
