# Supplementary material for: Identification of the hub and prognostic genes in liver hepatocellular carcinoma via bioinformatics analysis
Source: Front Mol Biosci. 2022 Sep 29;9:1000847. doi: 10.3389/fmolb.2022.1000847 (PMC9557295; doi:10.3389/fmolb.2022.1000847)
Supplement: Supplementary file 1 [file DataSheet1.ZIP › revised-Supplementary Materials Presentation/revised-Supplementary material.pdf]

Supplementary material

The number of Supplementary Figures: 4

The number of Supplementary Tables: 4

Supplementary Figure Legends

Supplementary Figure 1. The dendrogram of cluster in TCGA samples.

Supplementary Figure 2. Identification of key modules using WGCNA.
(A). The dendrogram of the clustered modules.
(B). Gene correlation scatter plot of the lightcyan module and purple module.
Left panel: lightcyan module. Right panel: purple module.

Supplementary Figure 3. GO-CC and GO-MF terms enrichment of genes in key module.
(A). GO-CC terms enrichment of genes in the turquoise module.
(B). GO-MF terms enrichment of genes in the turquoise module.

Supplementary Figure 4. Validation of the prognostic model.
(A). The ROC curve of the performance of the prognostic model at 1, 3 and 5 years in the training dataset.
(B). Heatmap of gene expression of prognostic genes in the training and test dataset.
Left panel: training dataset. Right panel: test dataset.
(C). The overall survival of detrimental prognostic genes in test dataset.

Supplementary Table

Supplementary Table 1. Detail information in GEO datasets.
Supplementary Table 2. Detail information of MCODE score.
Supplementary Table 3. Value of GS and MM in four hub genes.
Supplementary Table 4. All scores by different methods of hub genes

Supplementary Table 1. Detail information in GEO datasets

GEO accession	platform	Tumor	Normal
GSE84402	GPL570	14	14
GSE101685	GPL570	24	8
GSE101685	GPL16043	20	20
In total		58	42

Supplementary Table 2. Detail information of MCODE score

Module	nodes	edges	score
Modul1	57	2986	53.321
Modul2	10	76	8.444
Modul3	12	58	5.273
Modul4	5	20	5.000
Modul5	4	12	4

Supplementary Table 3. Value of GS and MM in four hub genes

Gene	GS	MM
AURKA	0.624440434	0.819895385
CCNB1	0.61699264	0.916708944
DLGAP5	0.609786183	0.906359812
NCAPG	0.39858859	0.838999704

Supplementary Table 4. All scores by different methods of hub genes

Gene	MCC	MNC	EPC	Degree	Closeness
AURKA	2.55E+53	60	106.542	120	93.86785714
CCNB1	2.55E+53	60	106.542	120	93.86785714
DLGAP5	2.55E+53	56	106.542	112	89.16785714
NCAPG	2.55E+53	56	106.542	114	89.91785714
