# Supplementary figures and images for: Identification of the hub and prognostic genes in liver hepatocellular carcinoma via bioinformatics analysis
Source: Front Mol Biosci. 2022 Sep 29;9:1000847. doi: 10.3389/fmolb.2022.1000847 (PMC9557295; doi:10.3389/fmolb.2022.1000847)

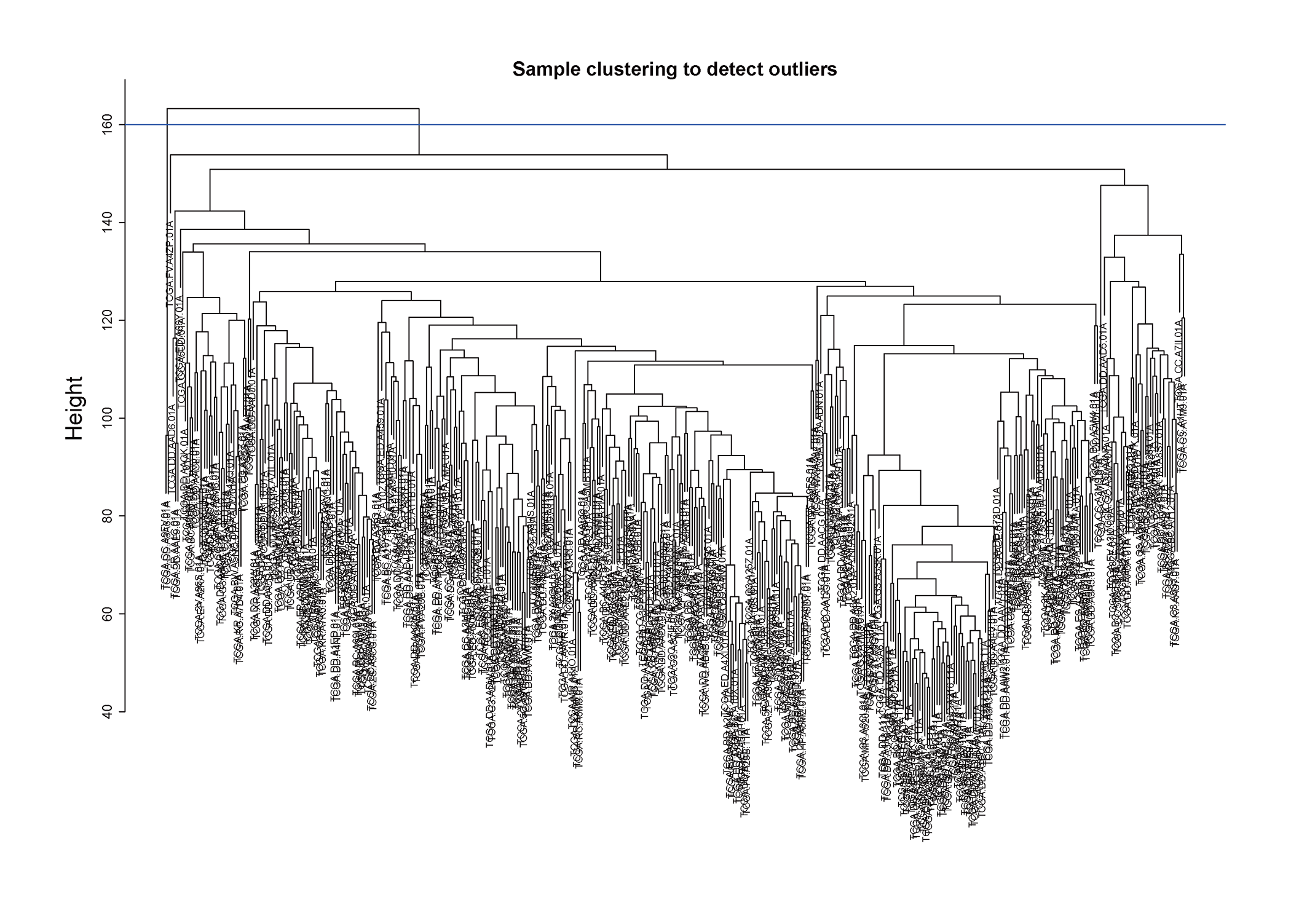

Supplement: Supplementary file 1 [file DataSheet1.ZIP › revised-Supplementary Materials Presentation/revised-Supplementary figure 1.tif]

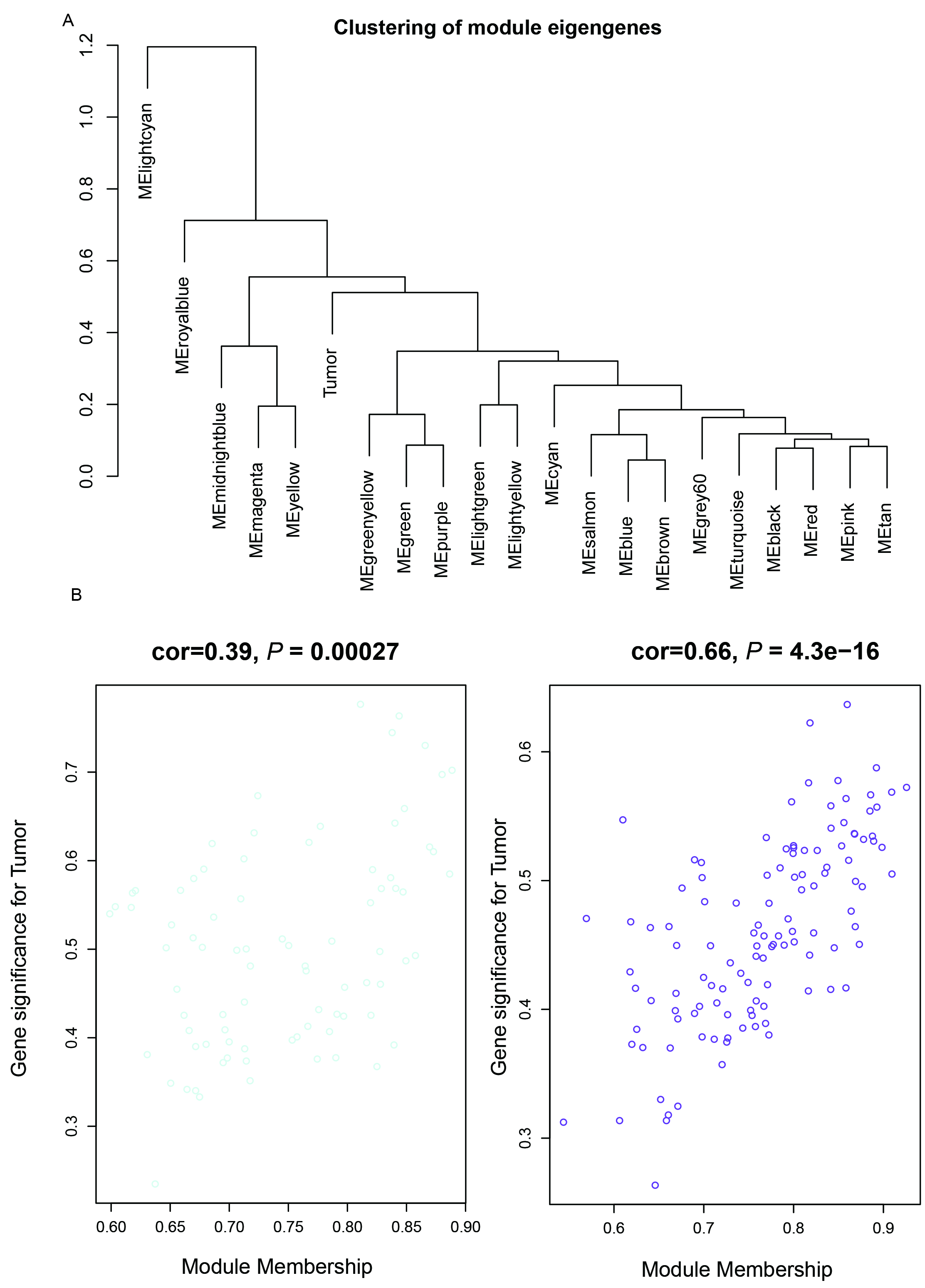

Supplement: Supplementary file 1 [file DataSheet1.ZIP › revised-Supplementary Materials Presentation/revised-Supplementary figure 2.tif]

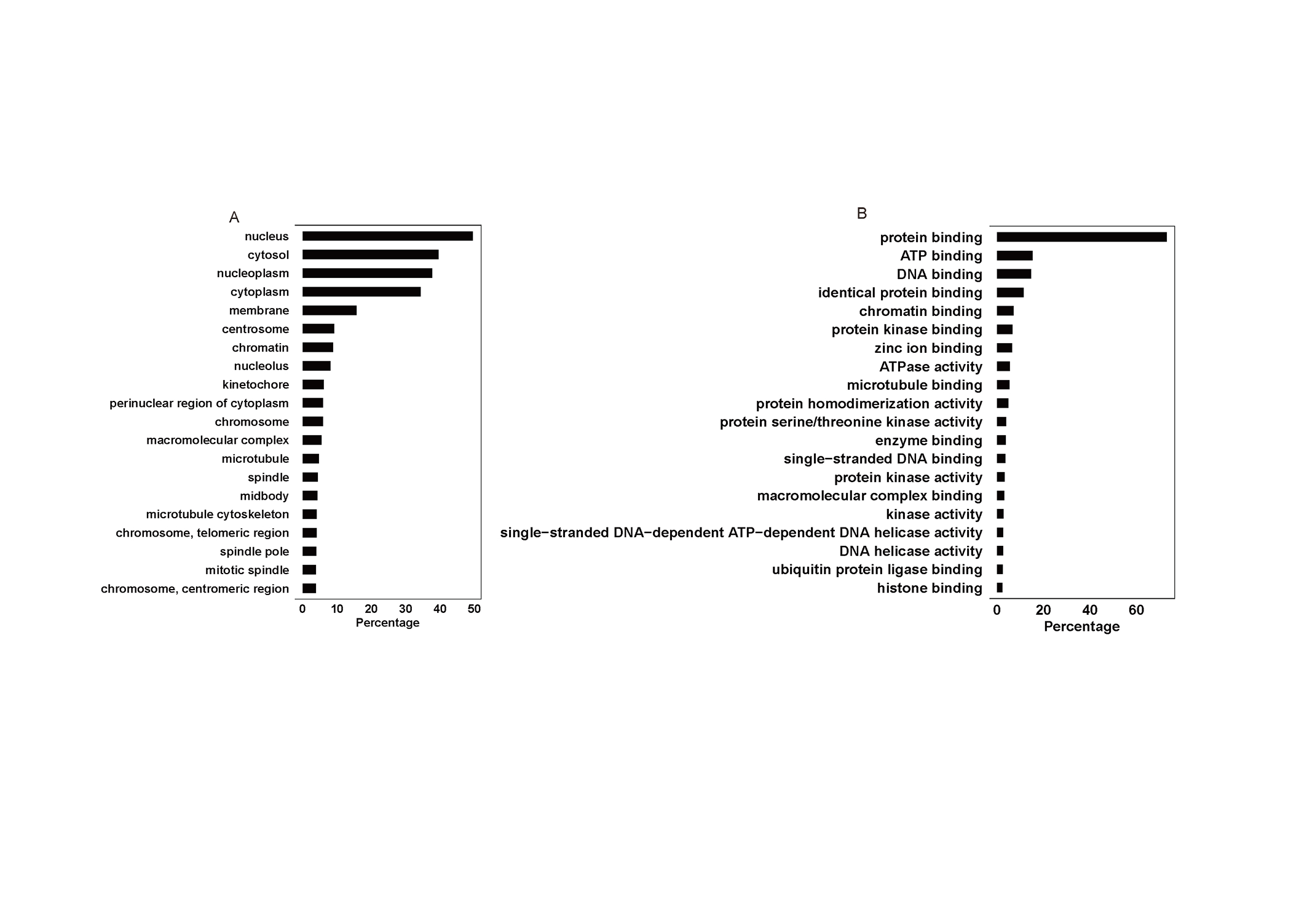

Supplement: Supplementary file 1 [file DataSheet1.ZIP › revised-Supplementary Materials Presentation/revised-Supplementary figure 3.tif]

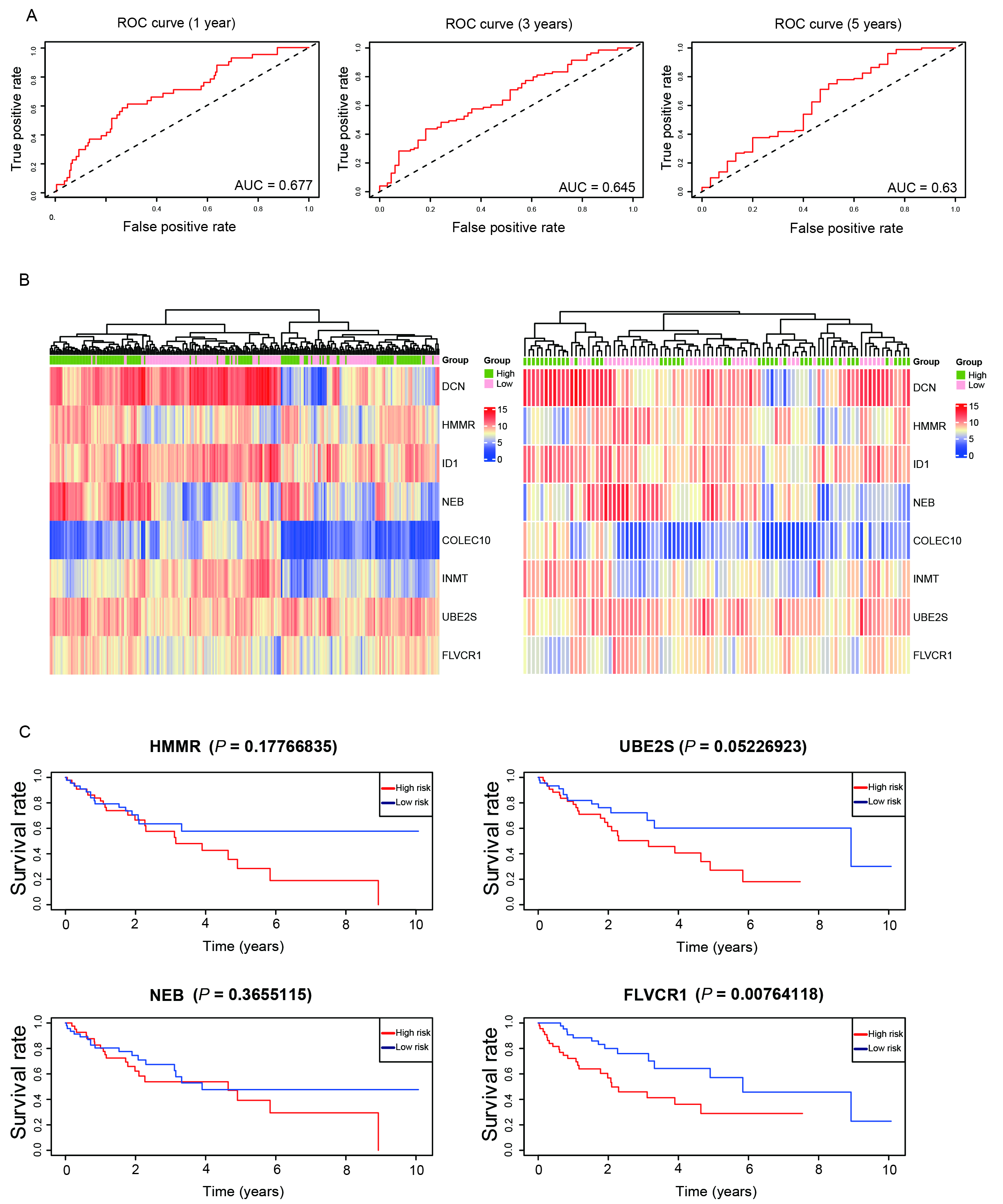

Supplement: Supplementary file 1 [file DataSheet1.ZIP › revised-Supplementary Materials Presentation/revised-Supplementary figure 4.tif]
